# Supplementary material for: Saccharomyces boulardii (CNCM I-745) improves intestinal damage in sepsis by remodeling intestinal flora structure
Source: Front Cell Infect Microbiol. 2025 Jul 31;15:1602792. doi: 10.3389/fcimb.2025.1602792 (PMC12350329; doi:10.3389/fcimb.2025.1602792)
Supplement: Supplementary file 1 [file DataSheet1.zip › summary/summary/8_Advanced_analyse/2_PICRUSt2_result/KEGG/Group.BW4_vs_AW4/PICRUSt2_KEGG_level3_Group.BW4_vs_AW4_diff.pdf]

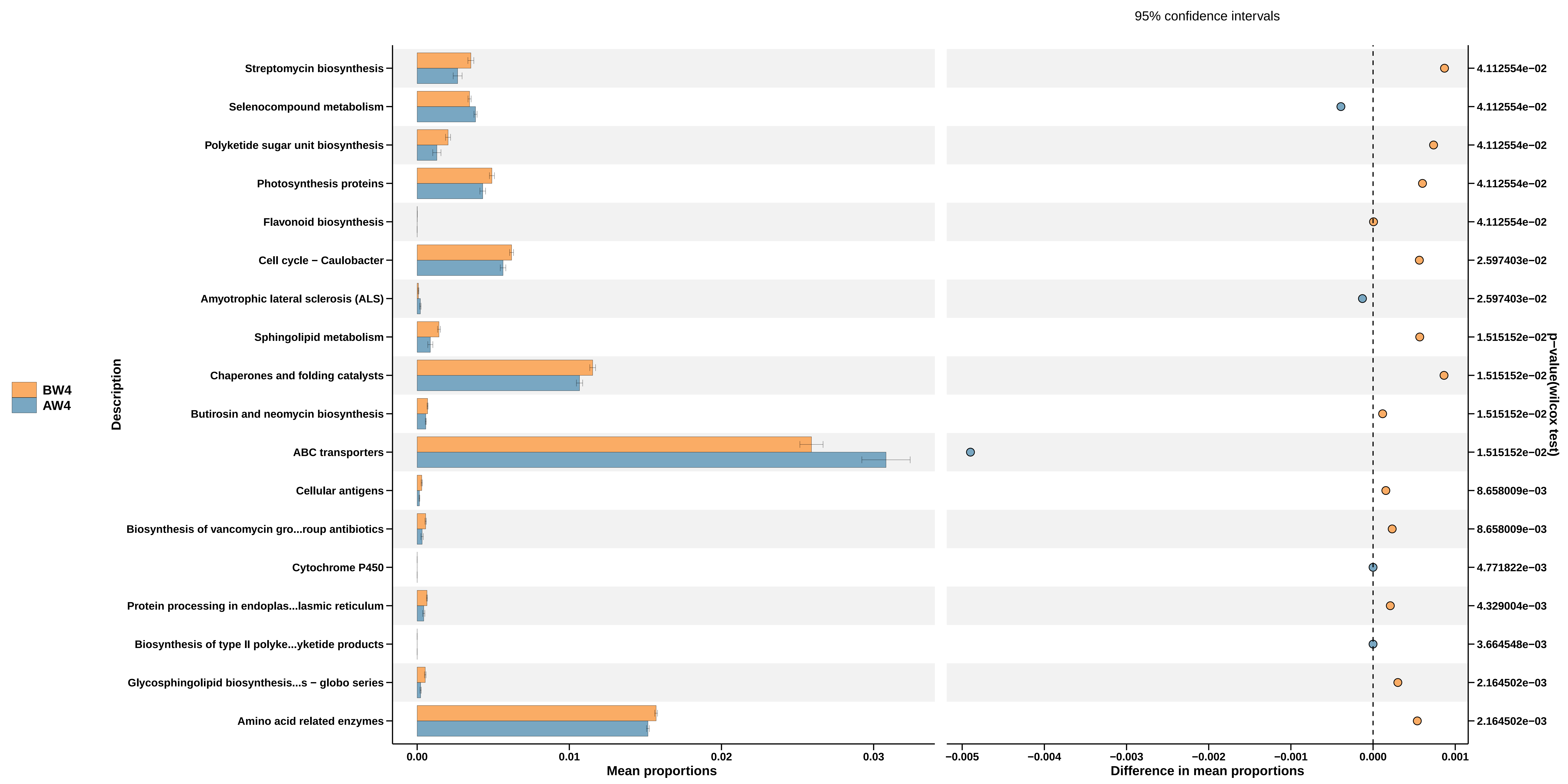
95% confidence intervals
BW4
AW4
Description
Streptomycin biosynthesis
Selenocompound metabolism
Polyketide sugar unit biosynthesis
Photosynthesis proteins
Flavonoid biosynthesis
Cell cycle − Caulobacter
Amyotrophic lateral sclerosis (ALS)
Sphingolipid metabolism
Chaperones and folding catalysts
Butirosin and neomycin biosynthesis
ABC transporters
Cellular antigens
Biosynthesis of vancomycin gro...roup antibiotics
Cytochrome P450
Protein processing in endoplas...lasmic reticulum
Biosynthesis of type II polyke...yketide products
Glycosphingolipid biosynthesis...s − globo series
Amino acid related enzymes
0.00
0.01
0.02
0.03
Mean proportions
−0.005
−0.004
−0.003
−0.002
−0.001
0.0000
0.001
Difference in mean proportions
4.112554e−02
4.112554e−02
4.112554e−02
4.112554e−02
4.112554e−02
2.597403e−02
2.597403e−02
1.515152e−02
1.515152e−02
1.515152e−02
1.515152e−02
8.658009e−03
8.658009e−03
4.771822e−03
4.329004e−03
3.664548e−03
2.164502e−03
2.164502e−03
p−value(wilcox test)
